# Gender Differences in Homicide of Neonates, Infants, and Children under 5 y in South Africa: Results from the Cross-Sectional 2009 National Child Homicide Study

**DOI:** 10.1371/journal.pmed.1002003

**Published:** 2016-04-26

**Authors:** Naeemah Abrahams, Shanaaz Mathews, Lorna J. Martin, Carl Lombard, Nadine Nannan, Rachel Jewkes

**Affiliations:** 1 Gender and Health Research Unit, South African Medical Research Council, Cape Town, South Africa; 2 Children’s Institute, University of Cape Town, Cape Town, South Africa; 3 Forensic Pathology Services, Western Cape Government, Cape Town, South Africa; 4 Division of Forensic Medicine and Toxicology, University of Cape Town, Cape Town, South Africa; 5 Biostatistics Unit, South African Medical Research Council, Cape Town, South Africa; 6 Burden of Disease Research Unit, South African Medical Research Council, Cape Town, South Africa; Makerere University Medical School, UGANDA

## Abstract

**Background:**

Homicide of children is a global problem. The under-5-y age group is the second largest homicide age group after 15–19 y olds, but has received little research attention. Understanding age and gender patterns is important for assisting with developing prevention interventions. Here we present an age and gender analysis of homicides among children under 5 y in South Africa from a national study that included a focus on neonaticide and infanticide.

**Methods and Findings:**

A retrospective national cross-sectional study was conducted using a random sample of 38 medico-legal laboratories operating in 2009 to identify homicides of children under 5 y. Child data were abstracted from the mortuary files and autopsy reports, and both child and perpetrator data data were collected from police interviews. We erred towards applying a conservative definition of homicide and excluded sudden infant death syndrome cases. We estimated that 454 (95% CI 366, 541) children under the age of 5 y were killed in South Africa in 2009. More than half (53.2%; 95% CI 46.7%, 59.5%) were neonates (0–28 d), and 74.4% (95% CI 69.3%, 78.9%) were infants (under 1 y), giving a neonaticide rate of 19.6 per 100,000 live births and an infanticide rate of 28.4 per 100,000 live births. The majority of the neonates died in the early neonatal period (0–6 d), and abandonment accounted for 84.9% (95% CI 81.5%, 87.8%) of all the neonates killed. Distinct age and gender patterns were found, with significantly fewer boy children killed in rural settings compared to urban settings (odds ratio 0.6; 95% CI 0.4, 0.9; *p* = 0.015). Abuse-related killings and evidence of sexual assault were more common among older girls than in all other age and gender groups. Mothers were identified as the perpetrators in all of the neonaticides and were the most common perpetrators overall (71.0%; 95% CI 63.9%, 77.2%). Abandoned neonates were mainly term babies, with a mean gestational age of 38 wk. We did not have information on abandonment motives for all newborns and did not know if babies were abandoned with the intention that they would die or with the hope that they would be found alive. We therefore considered all abandoned babies as homicides.

**Conclusions:**

Homicide of children is an extreme form or consequence of violence against children. This national study provides one of the first analyses of neonaticide and infanticide by age and gender and shows the failure of reproductive and mental health and social services to identify and help vulnerable mothers. Multi-sectoral prevention strategies are needed.

## Abstract 2

Jewkes and colleagues present a cross-sectional study that reveals levels of child homicide in South Africa. Identifying causes and vulnerable mothers will lead to prevention methods and strategies.

## Editors' Summary

**Background:**

Child mortality (death) is a global public health concern. In 2015, 5.9 million children (43 out of every 1,000 children born alive) died before their fifth birthday. Nearly half of these deaths occurred among neonates (babies 28 days old or younger); three-quarters of them occurred among infants (children less than one year old). Most of these deaths happened in resource-limited countries following delivery complications, infections, and other natural causes. Some, however, were homicides. Infanticide (the killing of a child under one year old) and neonaticide (the killing of a newborn within 24 hours or 28 days of birth, depending on the definition used) can be active (deliberate killing by, for example, suffocation) or passive (killing through nutritional, physical, or emotional neglect). Many infanticides and neonaticides are the result of abandonment—the mother leaves her infant or newborn in a place without care or protection, either with the intention of killing the child or with the hope that someone will find and care for him/her. Unwanted pregnancies are the most common cause of infanticide and neonaticide, but some infants are also killed because they are disabled or of the wrong gender—in some cultures male children are considered more socially valuable than female children.

**Why Was This Study Done?:**

To develop interventions to prevent child homicide, we need to understand when, where, and why these deaths occur. However, little research has been done on neonaticide and infanticide, particularly in resource-limited settings, because these deaths are often poorly identified and reported. In Africa, for example, although quantitative (numerical) research on child homicide is rare, Dar es Salaam, Tanzania, has one of the highest reported rates of neonaticide (27.7 homicides within 24 hours of birth per 100,000 live births); by comparison, a review of studies undertaken in the US, UK, and New Zealand reported incidences of infanticide and neonaticide ranging between 2.1 and 6.9 per 100,000 live births. Here, the researchers use data collected in a national cross-sectional mortuary-based study of child homicides committed in 2009 to investigate patterns among homicides of neonates, infants, and children under five years old in South Africa.

**What Did the Researchers Do and Find?:**

The researchers chose a random sample of 38 medico-legal laboratories (mortuaries where postmortem examinations were performed on all unnatural deaths) across South Africa. They used data extracted from mortuary files, autopsy reports, and police interviews to identify homicides of children under five years old. The researchers estimated that 454 children under five years old were killed in South Africa in 2009. More than half of the children who died were neonates, and nearly three-quarters were infants, giving a neonaticide rate (defined in this study as homicide within the first 28 days of life) of 19 per 100,000 live births and an infanticide rate of 28 per 100,000 live births. Most of the neonates died in the early neonatal period—only eight out of 241 neonates were more than six days old when they died. Abandonment accounted for 84.9% of neonaticides, and abandoned neonates were mainly term babies. Overall, there was no gender bias among child homicides, but 56% of homicides of boys took place in an urban setting, compared to 44% of homicides of girls. Finally, young mothers were the most common perpetrators of child homicide.

**What Do These Findings Mean?:**

These findings show that, in 2009, the rates of infanticide and neonaticide in South Africa were much higher than the rates previously reported for the US, UK, and New Zealand. The rates reported here are likely to be underestimates because it is often difficult to distinguish infanticide from other causes of death during infancy. Another important limitation of this study is the lack of data on the motive for abandonment of newborns. Importantly, however, these findings suggest that a child born in South Africa is at the highest risk of being killed during its first six days of life. This may mean that mothers who abandon their babies simply do not want a child, and suggests that reproductive, mental health, and social services are failing to identify and help vulnerable mothers in South Africa. The researchers suggest, therefore, that multi-sectoral prevention strategies are needed to reduce child homicides in South Africa. A pilot child death review system that has been initiated in South Africa to identify and describe patterns and causes of child death could assist in the development of such prevention strategies.

**Additional Information:**

This list of resources contains links that can be accessed when viewing the PDF on a device or via the online version of the article at http://dx.doi.org/10.1371/journal.pmed.1002003.

- This study is further discussed in a *PLOS Medicine* Perspective by Delan Devakumar and David Osrin
- The World Health Organization (WHO) provides information on child health and child mortality and global efforts to reduce child mortality and a factsheet on child maltreatment (available in several languages); a previous paper by the researchers on child homicide is available on the WHO website
- The United Nations Children’s Fund (UNICEF) works for children’s rights, survival, development, and protection around the world; its UNICEF Data website provides detailed statistics about child health and mortality; its 2014 report *Children in Danger: Act to End Violence against Children* provides detailed information about violence against children; *Hidden in Plain Sight* is a 2014 UNICEF statistical analysis of violence against children
- The international non-governmental organization Humanium, which works to stop violations of children’s rights worldwide, has an article on infanticide (in several languages)
- Child Trends is a US not-for-profit organization that aims to improve the lives and prospects of children and youth in the US by conducting research; it provides up-to-date information about infanticide in the US
- Information on the South African pilot child death review system is available; a guide on the child death review system in the UK for parents and carers is available

## Introduction

The number of children killed in a country is a proxy indicator of the effectiveness of the child protection system [1,2]; homicides of children under 5 y old, in particular, also reflect a failure in maternal and child health service provision. Many countries, particularly low- and middle-income countries, do not often collect or analyze data on homicides of children, although infant mortality is viewed as a key indicator for the development and health status of a country [3]. The 2014 report on violence against children published by the UNICEF presented homicide estimates, using mostly country administrative data (vital registration data), and cautioned about the validity of regional and country homicide estimates. The report concluded, however, that the under-5-y age group has the second largest number of homicides among children 0–19 y old, after the 15–19 y age group [4]. This pattern was shown in the South African National Child Homicide Study, with 39.6% of all child homicide deaths among the under-5-y group (41.0% among 15–17 y olds) [5].

Nearly half a century ago, Resnick [6] documented and described the homicide vulnerability of children under 5 y old. His work focused on infanticide (killing of a child under 1 y—see Box 1 for explanation of terms), and he introduced the term neonaticide, which he defined as the homicide of a newborn within the first 24 h after birth. Although child homicide has not received much research attention, studies mainly from high-income settings have shown that children under 1 y have a four times greater risk of being killed than children in all other age groups, with the highest risk in the first day of life [7,8]. A review of 40 y of research on both infanticide and neonaticide showed the incidence from seven studies—all from developed settings (US, UK, and New Zealand)—to range from 2.1 to 6.9 per 100,000 live births [7]. Very little epidemiological data are available on neonaticide and infanticide outside of high-income countries, but, more recently, a 13-y review from Malaysia reported an infanticide rate ranging from 4.8 to 9.1 per 100,000 live births [9]. There is little published literature on infant abandonment/infanticide in Africa. Most of the papers have been qualitative [10,11], although one from Ghana asserted that nearly 15% of deaths under the age of 3 mo could be linked to infanticide practices [12]. A survey of violent deaths in the city of Dar es Salaam, Tanzania [13], estimated a rate of neonaticide (within 24 h of birth) of 27.7 per 100,000 live births, which is one of the highest reported. The only South African child homicide study was conducted using data on injury-related deaths collected through the National Injury Mortality Surveillance System (NIMSS). Prinsloo et al. [14] performed a secondary analysis on data on children 0–14 y from four South African cities where NIMSS coverage of data collection was considered complete for 2001 to 2005. They found the pattern of child homicides to be similar to the global pattern, with higher rates reported among 0–4 y olds and among boys aged 10–14 y. This study also found that 10% of the homicides were related to baby abandonment [14].

**Box 1. Definitions of Terms**

Neonaticide: killing of a child within the first 28 d of life (newborn). Rates are calculated per 100,000 live births. (Many studies define neonaticide as killing of a newborn within the first 24 h after birth.)

Early neonaticide: killing of a newborn within 0–6 d
Late neonaticide: killing of a newborn within 7–28 d
Active neonaticide: deliberate killing
Passive neonaticide: death due to negligence directly following birth

Infanticide: killing of a child under 1 y (infant). Rates are calculated per 100,000 live births. (In some countries infanticide refers specifically to killing of an infant by the mother, but in this paper we are not restricting infanticide to deaths caused by mothers.)

Active infanticide: deliberate killing
Passive infanticide: death due to medical, nutritional, physical, and emotional neglect

Filicide: killing of a child by a parent

Maternal filicide: killing of child by a mother
Paternal filicide: killing of a child by a father

Abandonment: the leaving of an infant or newborn in a place without care, protection, or supervision

Concealed pregnancy: a legal term for a surreptitious birth followed by abandoning the newborn

Family murder: a child homicide where other family members are killed in the same incident. A parent or a parent’s boyfriend/girlfriend is often the perpetrator.

Decreasing child mortality was one of the key Millennium Development Goals and will continue as a priority in the new Sustainable Development Goals, but South Africa has been struggling to attain its goal; child mortality has remained above the global rate for the last 15 y. In 2009, the neonatal mortality rate was 14 per 1,000 live births, the infant mortality rate was 39 per 1,000 live births, and the under-5-y mortality rate was 56 per 1,000 live births [15]. Child homicide is often not considered a priority against competing public health problems contributing to these statistics. In addition, there has been an opinion in the child homicide field that the context of child homicide is different in low- and middle-income settings. For example, in a review of child homicides by mothers (maternal filicide) [16], data from developing settings were excluded because of “the likelihood that different factors for infant homicides exist in undeveloped countries…[due to] varying cultural, legal and/or economic factors…”[16].

One of the problems with neonaticide and infanticide research is that there is poor routine identification, reporting, and recording of these deaths. Separate reporting and investigation systems that do not always interface compound the difficulties, as well as the fact that such deaths are easily hidden or disguised as natural deaths, especially in less resourced settings [7,17]. Even in high-income countries, different studies use different age categorizations in their analyses, which makes comparisons difficult. Most studies measuring neonaticide measure deaths that occur within the first 24 h of life [7], but one North Carolina study included newborns 0–4 d old [18]. Both of these age groupings differ substantially from the standard neonatal period of 0–28 d used by epidemiologists and public health researchers [3] and that we also use in this study.

The first South African national mortuary-based study of child and adult female homicides focused on those committed in 2009 [5]. It confirmed a wide variation in the age-specific incidence of child homicide, with a bimodal pattern of higher incidence in the youngest and the oldest child age groups [5]. This study provides us with an opportunity to further analyze the gender differences in the under-5-y age group, and in this paper we separate neonaticide and infanticide and use live births as the denominator to calculate the mortality rates within infancy. We also present an analysis of the characteristics of homicides in children under 5 y by gender, as identifying factors associated with these homicides is important for prevention interventions.

## Methods

Ethical approval for the study was granted by the Ethics Committee of the South African Medical Research Council (EC009-021), and further approval and access to data was obtained from the National Department of Health, the provincial Forensic Pathology Services, the National Prosecuting Authority and the South African Police Service.

We conducted a national retrospective mortuary-based study of child homicides. A postmortem examination is performed on all unnatural deaths, a statutory requirement of the Inquests Act 58 of 1959 [19], and we identified and included child homicides presenting to medico-legal laboratories (MLLs) between 1 January 2009 and 31 December 2009. All operating mortuaries in the country (123 were operating in 2009) were stratified into three groups based on the number of autopsies performed per year. The strata were as follows: small, <500 autopsies; medium, 500–1,499 autopsies; and large, ≥1,500 autopsies per year. We preselected to draw a random sample from each of the three groups using the following sampling fractions: 55.6% large mortuaries (5 of 9), 39.4% medium mortuaries (13 of 33), and 24.7% small mortuaries (20 of 81). We wanted to ensure the sample was representative and included both small rural mortuaries and large ones attached to medical schools. The methodology of the broader study is described in detail elsewhere [5,20]. The sample size calculation for the study was based on the child homicide data from the NIMSS for the period 2001–2004 [14]. We took into account that the NIMSS survey covered 40% of the homicides in South Africa and that the larger dataset included 19 y olds in the oldest child age group (15–19 y). We also took into account the decline in homicides in South Africa over the last 10 y overall. We estimated that we required a sample of 800 child homicide cases to obtain national representative estimates with acceptable precision, and we did not aim to do provincial estimates. In addition, we used the same sampling frame (MLLs) as the first national female homicide study [21]. All child homicide cases found in a mortuary for 2009 were included in the study.

We used a series of processes to identify child homicide cases. The first process was to identify cases of non-natural deaths from the death registers at the sampled MLLs, where cause of death and age are documented. At this stage, we excluded non-natural deaths where the death was clearly identified as an accident in the death register, i.e., motor vehicle accident. We included cases where non-natural deaths had no clear definitive information about the cause of death. The second process was to review the mortuary files and postmortem reports of identified cases to validate the age and cause of death. If a case was included, we abstracted data on demographics, cause of death, and police case identification details. The third process was identifying and contacting the police investigating officer for each case, and an interview was done to verify age and cause of death and to obtain information on the circumstances of the death, the perpetrator (or main suspect), the victim–perpetrator (or suspect) relationship, whether child abuse was confirmed or suspected, and, for abandoned babies, details such as where bodies were found. All of the above was captured on a data capture sheet.

All non-natural deaths such as deaths by drowning, fire, poisoning, and falls were reviewed for inclusion, and only if both the pathology findings and data on the circumstances of the death from the police interview indicated accidental or unintentional death did we exclude them. We excluded stillbirths and sudden infant death syndrome (SIDS) cases as identified in the pathology report but included cases labeled as “concealed pregnancy” in the postmortem records, a legal term used in South Africa for a surreptitious birth followed by abandoning the newborn. Such concealed pregnancies are considered a crime in South Africa when the death that follows is linked to the act of being abandoned. Such cases were included only if there was evidence of a live birth of a viable newborn per post-mortem examination. We collected additional data on mass, length, and estimated gestational age. We considered fatal child abuse and neglect as a subset of child homicide, as described previously [5]. This subset included cases of abandoned newborns and cases with evidence of sexual violation that was confirmed by a forensic examination and police investigation.

Data were analyzed using Stata version 13. The data analysis took into account the cluster sample survey design, and weights were applied to account for the selection probabilities of MLLs within survey strata. The survey included homicides of all children up to 18 y, and we used domain analysis, which allowed us to analyze the subpopulations of interest, i.e., the subgroup children under 5 y and the subgroup of abandoned neonates. Descriptive statistics (survey means and proportions) were calculated, as well as standard errors and 95% confidence intervals. Categorical variables were compared using Pearson’s χ^2^ test, and standard errors and 95% confidence intervals were calculated using methods for complex sample surveys (Taylor linearization). We used a regression analysis to test differences between means for continuous variables and logistic regression to test differences between proportions for categorical variables. We were particularly interested in testing whether the age and sex of children were associated with a number of outcomes (urban/rural status, child abuse, and perpetrator age), and the models were tested with interaction terms. We stratified the ages of the children into early neonates (0–6 d), late neonates (7–28 d), 1–11 mo, and 1–4 y to do age-specific analysis. Numbers of live births derived from the 2011 census were used to calculate age-specific mortality occurring in the first year of life. The mortality rates of children aged 1–4 y old were calculated using the alternative mid-year population estimates produced by the Centre for Actuarial Research [22]. We also performed a subpopulation analysis for abandoned neonates and looked at differences between boys and girls.

## Results

We estimate a total of 454 (95% CI 366, 541) children under the age of 5 y were killed in 2009 in South Africa. More than half (53.2%; 95% CI 46.7%, 59.5%) were neonates, and 74.4% (95% CI 69.3%, 78.9%) were infants under 1 y (see Table 1). The overwhelming majority of the neonates died in the early neonatal period (only eight neonates were older than 6 d). The estimated rate of early neonaticide was 19.6 per 100,000 live births (95% CI 14.6, 23.1). Lower rates were observed in older age groups, and this trend was observed in both girls and boys.

**Table 1:** Estimated age-specific homicide rates per 100,000 live births for children under 5 y.

Age Group	Number of Homicides (95% CI)	Percentage of All Homicides in Children under 5 y (95% CI)	Homicide Rate per 100,000 (95% CI)		
			All	Boys	Girls
Early neonates (0–6 d)*	233 (173, 294)	51.4 (45.1, 57.6)	19.6 (14.6, 23.1)	19.4 (13.8, 25.2)	18.8 (12.2, 25.3)
Late neonates (6–28 d)*	8 (2, 14)	1.8 (0.8, 3.8)	0.7 (0.2, 1.2)	0.7 (0.0, 1.5)	0.6 (0.0, 1.5)
1–11 mo*	96 (67, 126)	21.2 (17.2, 25.8)	8.1 (5.6,10.6)	8.0 (5.3, 10.7)	8.0 (3.7, 12.2)
Infants (<1 y)*	337 (259, 417)	74.4 (69.3, 78.9)	28.3 (21.8, 35.1)	28.1 (22.9, 33.5)	27.5 (18.3, 36.6)
1–4 y**	116 (95, 137)	25.5 (21.0, 30.6)	1.0 (0.0, 3.2)	2.8 (1.9, 3.4)	2.6 (1.8, 3.4)

Live births for 2009 for South Africa: boys, 608,248; girls, 596,164. Mid-year population for children 1–4 y: boys, 2,182,408; girls, 2,145,536.

*Rates based on live births.

**Rates based on the mid-year population.

Demographics of the victim, perpetrator and homicide by sex and age groups are presented in Table 2. Overall urban/rural differences were found for boys versus girls (*p* = 0.01), with 56% of male homicides taking place in an urban setting compared to 44% of female homicides. Overall, significantly fewer boy children were killed in rural settings than in urban settings (odds ratio 0.61; 95% CI 0.41, 0.90; *p* = 0.015), but no further significant interactions were found between age, sex, and location. Mothers were identified or suspected as the perpetrators in nearly two-thirds of the homicides (71.0%) and in all of the neonaticides. Few children were killed by strangers (2.5%), and one in five of the children were killed in the context of a family murder (with one or more other family members also killed). Suicide following the killing of a child was not common—only 2.7% of the perpetrators committed suicide, and this was more often a father than any other perpetrator group.

**Table 2:** Characteristics of homicides in children under 5 y by age group and sex.

Characteristic	Boys			Girls			All Children <5 y
	Neonates (0–28 d)	1–11 mo	1–4 y	Neonates (0–28 d)	1–11 mo	1–4 y	
**All homicides by sex**	51.4% (40.8%, 61.9%)	21.7% (15.4%, 29.7%)	26.8% (20.8%, 33.7%)	52.6% (42.3%, 62.7%)	21.8% (14.9%, 30.8%)	25.6% (17.9%, 35.0%)	
**Urban/rural location**							
Urban	72.5% (64.7%, 79.3%)	50.4% (28.9%, 71.6%)	66.5% (51.8%, 78.5%)	57.1% (43.7%, 69.4%)	60.1% (41.1%, 76.4%)	44.5% (25.7%, 64.9%)	59.8% (54.2%, 65.2%)
Small town/rural	27.5% (20.7%, 35.3%)	49.6% (28.3%, 71.0%)	33.5% (21.5%, 48.1%)	42.9% (30.5%, 56.7%)	39.9% (23.5%, 58.9%)	55.5% (35.0%, 74.3%)	40.2% (34.9%, 45.8%)
**Perpetrator was mother**	100%	47.7% (30.7%, 65.2%)	38.3% (22.1%, 57.6%)	100%	50.2% (29.5%, 0.9%)	19.4% (10.1%, 34.0%)	71.0% (63.9%, 77.2%)
**Perpetrator mean age (years)**	24.5 (21.0, 27.9)	27.9 (24.4, 31.4)	29.9 (25.5, 34.4)	24.4 (21.9, 26.9)	31.2 (26.8, 35.6)	28.8 (25.9, 31.7)	27.7 (26.0, 29.3)
**Perpetrator <20 y**	24.9% (10.2%, 49.4%)	12.0% (2.9%, 38.1%)	13.7% (5.3%, 31.0%)	20.5% (7.1%, 46.5%)	9.4% (1.9%, 36.0%)	4.5% (0.7%, 22.8%)	14.7% (8.5%, 24.2%)
**Perpetrator employed**	14.6% (8.7%, 23.3%)	13.9% (5.4%, 31.2%)	14.9% (6.7%, 30.2%)	0%	16.5% (5.4%, 40.8%)	37.3% (24.8%, 51.8%)	14.0% (10.8%, 17.9%)
**Child abuse/neglect**	98.5% (91.7%, 99.7%)	52.8% (31.7%, 72.9%)	49.6% (28.5%, 70.9%)	100%	70.7% (57.1%, 81.4%)	61.1% (44.1%, 75.8%)	80.0% (75.7%, 83.6%)
**Rape suspected**	0%	0%	3.0% (0.05%, 15.7%)	0%	0%	25.4% (13.4%, 42.9%)	3.5% (1.7%, 7.1%)
**Family murder**	0%	28.1% (9.6%, 58.9%)	43.1% (26.3%, 61.7%)	0%	18.7% (3.7%, 58.2%)	59.6% (30.2%, 83.4%)	19.8% (12.2%, 30.5%)
**Location where body was found**							
At victim’s home	6.7% (3.2%, 13.5%)	70.9% (56.5%, 82.0%)	68.7% (52.1%, 81.6%)	19.0% (10.1%, 32.7%)	60.5% (43.9%, 75.0%)	59.4% (45.3%, 72.0%)	37.1% (32.1%, 42.3%)
Other home	15.9% (8.1%, 29.0%)	12.0% (4.2%, 29.3%)	16.9% (7.8%, 33.0%)	3.7% (1.2%, 11.3%)	33.1% (20.1%, 49.3%)	17.9% (9.4%, 31.2%)	13.9% (9. 5%, 19.9%)
Public space	41.4% (28.0%, 56.3%)	8.8% (2.6%, 25.6%)	10.1% (3.8%, 24.3%)	57.2% (44.9%, 68.3%)	6.3% (1.2%, 26.9%)	18.2% (8.5%, 34.7%)	31.8% (25.5%, 38.9%)
Other	2.1% (0.4%, 9.4%)	8.3% (2.7%, 2.6%)	0%	0%	0%	4.5% (0.8%, 19.8%)	15.3% (11.8%, 19.5%)
Unknown	33.6% (25.6%, 42.6%)	0%	4.1% (0.8%, 18.2%)	20.0% (12.4%, 30.6%)	0%	0%	2.0% (0.8%, 4.7%)
**Cause of death**							
Concealed pregnancy	69.5% (58.4%, 78.6%)	0%	0%	71.8% (63.3%, 79.0%)	0%	0%	38.3% (32.5%, 44.5%)
Blunt force	11.3% (7.1%, 17.5%)	44.0% (31.1%, 57.7%)	31.6% (18.5%, 48.3%)	7.9% (3.4%, 17.0%)	48.3% (32.6%, 64.3%)	26.7% (15.2%, 42.5%)	22.1% (17.6%, 27.5%)
Strangulation	5.0% (2.1%, 11.5%)	22.7% (11.9%, 39.0%)	26.7% (15.1%, 42.6%)	16.5% (11.7%, 22.7%)	5.2% (1.0%, 3.6%)	21.0% (10.6%, 37.1%)	14.6% (11.5%, 18.3%)
Gunshot	0%	16.6% (8.9%, 28.7%)	10.9% (4.4%, 24.5%)	0%	8.5% (1.9%, 30.0%)	19.4% (8.1%, 39.7%)	6.5% (4.0%, 10.3%)
Stab	0%	0%	3.0% (0.5%, 15.7%)	0%	5.2% (0.9%, 23.6%)	19.4% (10.1%, 34.0%)	3.3% (1.7%, 6.3%)
Asphyxiation	0%	0%	0%	0%	5.2% (0.9%, 23.6%)	0%	0.5% (0.1%, 2.6%)
Fire death	0%	8.3% (2.7%, 22.6%)	16.5% (6.0%, 37.9%)	0%	0%	4.5% (0.8%, 19.8%)	3.6% (1.8%, 7.0%)
Drowning	3.5% (1.0%, 11.3%)	0%	0%	0%	0%	0%	0.9% (0.3%, 2.9%)
Multiple injuries	3.5% (1.2%, 9.6%)	0%	4.1% (0.8%, 18.2%)	0%	0%	4.5% (0.8%, 19.8%)	2.0% (0.9%, 4.1%)
Undetermined	3.5% (1.2%, 9.6%)	0%	0%	3.7% (1.2%, 11.3%)	22.2% (12.1%, 37.3%)	0%	4.1% (2.2%, 7.5%)
Other	3.5% (1.2%, 9.5%)	8.3% (2.0%, 28.1%)	7.1% (2.1%, 21.1%)	0%	5.2% (1.0%, 21.9%)	4.5% (0.8%, 19.8%)	3.8% (1.8%, 7.6%)
**Perpetrator convicted**	8.2% (4.4%, 14.9%)	13.9% (5.4%, 31.2%)	30.5% (18.4%, 46.2%)	6.5% (2.6%, 15.1%)	13.7% (7.6%, 25.8%)	47.8% (29.7%, 66.5%)	16.8% (12.5%, 22.0%)

Data are given as percent (95% CI) or mean (95% CI). In all, 50.6% (95% CI 46.3%, 54.9%) of the homicides were in boys, and 49.4% (95% CI 45.0%, 53.7%) were in girls.

The age of the perpetrator was known in less than 50% of the cases, and the mean age of the perpetrator was 27.7 y (95% CI 26.0, 29.3), with a significantly lower mean age if the perpetrator was a mother (24.8; 95% CI 23.1, 26.7) compared to other perpetrators (32.8; 95% CI 29.6, 36.0). Employment status of the perpetrator was known for only 53.8% of the cases. Similar levels of employment were reported among perpetrators of boy homicides across age groups; none of the perpetrators who killed newborn girls were employed, and higher levels of employment were reported among perpetrators who killed older girls. Almost all of the neonatal homicides were identified as child abuse/neglect cases. Although higher levels of abuse were reported in older girls (1–11 mo and 1–4 y) than older boys, in testing of interaction terms, the age and the sex of the victims were not found to have modifying effects on abuse. Evidence of sexual assault was found in both sexes but only in the older children (1–4 y), with a higher proportion of sexual violence found for girl homicides than for boy homicides (25.4% versus 3.0%; *p* < 0.001). The child’s home and public spaces were the most common places where the bodies were found, with neonates more commonly found in public spaces. Cause of death also differed by age group and sex. Concealed pregnancy was the most common cause of death overall (38.3%), followed by blunt force (22.1%). Blunt force was the most common cause of death in the older age groups (1–11 mo and 1–4 y) for both boys and girls, followed by strangulation, except for girls 1–11 mo old, for whom an undetermined cause of death was the second most common category (22.2%). In only 16.8% of the homicides in children under 5 y was the perpetrator convicted, and perpetrator convictions were more common in homicides of older children.

Table 3 presents the subgroup analysis for neonaticides involving abandonment, which accounted for 84.9% (95% CI 81.5%, 87.8%) of all neonaticides. Slightly more boys were abandoned (51.8%; 95% CI 39.5%, 63.8%) than girls (48.2%; 95% CI 34.1%, 60.5%), and significantly more abandoned neonates were found in urban settings than in rural settings (64.7% versus 35.2%; *p* = 0.007). The abandoned neonates were commonly discovered in garbage and refuse dumps and public spaces. The mean mass of the abandoned neonates was above 2,700 g, and the mean gestational age was 38 wk. More than 60% (61.5%) were estimated to have a gestational age of at least 40 wk, and only 7.0% were estimated to have a gestational age of 32 wk or less.

**Table 3:** Characteristics of neonaticides involving abandonment.

Characteristic	All Abandoned Neonates	Abandoned Boys	Abandoned Girls	*p-*Value
**Estimated number of cases**	205 (156, 254)	100 (67, 133)	93 (57, 129)	0.85
**Urban/rural location**				0.18
Urban	64.7% (56.3%, 72.3%)	71.0% (62.8%, 78.0%)	61.5% (47.5%, 73.8%)	
Small town/rural	35.2% (27.6%, 43.6%)	29.0% (22.0%, 37.2%)	38.5% (26.2%, 52.5%)	
**Location where body was found**				0.13
Garbage and refuse dumps	32.1% (22.3%, 43.7%)	33.3% (22.7%, 45.8%)	30.8% (17.8%, 47.8%)	
Public space/open veld	52.4% (40.9%, 63.7%)	58.1% (46.2%, 69.1%)	46.3% (30.3%, 63.4%)	
Toilet/sewage systems	5.8% (2.4%, 13.2%)	6.6% (2.1%, 18.4%)	5.0% (1.6%, 15.1%)	
Lake/river/dam	5.8% (2.4%, 13.2%)	2.0% (0.4%, 11.3%)	9.8% (4.8%, 18.7%)	
Buried in shallow grave	3.9% (1.1%, 12.8%)	0	8.0% (2.3%, 24.2%)	
**Mean mass (grams)** *	2,752 (2,604, 2,901)	2,873 (2,577, 3,168)	2,700 (2,487, 2,913)	0.39
**Mean gestational age (weeks)** *	38.1 (37.2, 38.9)	38.6 (37.8, 39.4)	38.5 (37.7, 39.2)	0.84
**Mean age of perpetrator (years)** *	23.5 (21.3, 25.8)	22.5 (19.6, 25.4)	25.6 (22.3, 28.9)	0.14

Data are given as *n* (95% CI), percent (95% CI), or mean (95% CI).

*Data not available for all cases.

The mean age of the perpetrators of neonaticides involving abandonment was 23.5 y, and these perpetrators were younger than the perpetrators of neonaticides not involving abandonment (29.2 y; 95% CI 27.5, 30.9) and the perpetrators of non-neonate homicides (30.9 y; 95% CI 30.0, 31.7; *p* < 0.001). No differences between boy and girl abandoned neonates were found for any of the characteristics in Table 3. Nearly two-thirds of the neonaticides that involved abandonment (73.6%) occurred in two provinces (Gauteng [43.1%] and KwaZulu-Natal [30.5%]) where 40% of the live births occur.

## Discussion

This is, to our knowledge, the first national study of homicides including neonaticide and infanticide in South Africa. The rates we estimated are among the highest reported rates for neonaticide (19.6 per 100,000 live births) and infanticide (28.4 per 100,000 live births), surpassed only by the estimate for Dar es Salaam (27.7 per 100,000 live births) [13], and are much higher than those reported in developed settings [7]. Homicide is not the most common cause of death among children under 5 y: the Second National Burden of Disease Study for South Africa found that injuries accounted for only 4.5% of all deaths in children under 5 y (the study did not separate homicides from all injury deaths), with HIV/AIDS, neonatal causes, and diarrheal disease among the leading causes of death [23]. Our study shows that the first 6 d of life are the time point of highest risk for being killed in South Africa among children under 5 y, with the risk declining thereafter. This suggests that there is a particularly high rate of unwanted pregnancy going to term, which is remarkable in a country that has one of the most liberal abortion laws in the world and reasonably good contraception services. It points primarily to a failure of maternal and reproductive health services. Research among women who were denied abortions in Cape Town showed that despite the law, there are numerous barriers that women encounter when seeking a legal abortion in the public sector [24]. Advanced gestational age and lack of trained staff to do second trimester abortion were common barriers, and women ended up seeking abortion outside of the legal system or continued with the unwanted pregnancy. Although our study did not explore the relationship between use of abortion services and neonaticide, the high rate of neonaticide suggests women with unwanted pregnancies are unable to terminate them, and neither are they considering adoption. Our findings point to the need for interventions within maternity services, and future research should explore the development of interventions such as option counselling, which may include providing information on how to give children up for adoption.

We found no difference in the rate of child homicide for girls and boys overall, and the analysis of neonaticides involving abandonment showed no gender differences. However, our study showed a 40% lower likelihood for boys to be killed in rural settings than in urban settings. This may reflect a lesser propensity to kill boys in more traditional areas in which a son preference is strong. Gender differences in the killing of children are commonly found in societies where a strong son preference is prevalent. This has been suggested as the main reason for the abnormally low sex ratios and the missing girl children in India and China [25]. In addition, greater acceptability and support for unwanted pregnancies may be more common in rural settings. We need to better understand differences in motives and socio-economic circumstances, as such understanding will assist in the development of prevention interventions.

We found a higher number of older girls (1–4 y) than older boys (1–4 y) killed in circumstances of child abuse, and girls were more likely to have sexual violence identified as part of the killing. Some of the estimates are based on small numbers, and this prevented us from doing more detailed analysis of differences between the age groups for boys and girls.

Our findings of mothers as the most common perpetrators and their young mean age are similar to the literature across the globe [9,16,26–28]. Two reviews [26,27] showed that mothers’ risk of perpetration was associated with economic stress, unemployment, younger age, limited education, social isolation, mental illness, substance abuse, and being victims of intimate partner violence. Most studies do not fully explore the direct and indirect role men have in women’s motives to kill their young children, although fear of abandonment by the male partner, lack of financial support, and having fragile relationships with the father have been reported [7,26,29,30]. An earlier study showed that men sometimes have a role in assisting women to source illegal abortions in South Africa, pointing to the possibility that deciding to abandon a newborn might also sometimes be a joint decision [31]. Studies have shown that denial of paternity and lack of awareness of the pregnancy in the social environment are among the variables most associated with neonaticide [29].

It has been suggested that the risk of child homicide is a product of a constellation of risk factors (some discussed above) [7,30]. Although the mental health of mothers who kill newborns and infants has been reported on extensively, a 40-y review of research on neonaticide has shown that women are usually not mentally ill when they kill their newborn, and suicide rarely follows the event [7,30]. Our study was not able to assess the mental state of the mothers who killed their children but qualitative studies have found that women who conceal their pregnancies and abandon their babies just do not want them. They also often have poor coping and problem-solving skills and are emotionally immature [26,32]. It is clear that the context of homicides of young children is still not fully understood, and further research is needed, in particular, better understanding of the unmet reproductive health needs of women who abandon term babies. Such unmet needs are a concern in a country where we consider ourselves to have reasonably good sexual education, contraception, and abortion services. In addition, we also need to understand the risk factors for child homicide across the different age groups, as interventions will differ. We have shown previously the role of child abuse in children under 5 y, the role of sexual homicide in older girls (15–17 y), and how homicide in older boys (15–17 y) mimics adult homicide [5]. South Africa is a country with both first- and third-world indices for health and wealth, resulting in vast inequalities [33] and high levels of violence [34], and although poverty has been identified as a risk factor for both neonaticide and infanticide in developed settings, we were not able to determine its role in our study as information on children’s home environment was not well recorded in the police data. We used perpetrator employment status as an indicator and found lower levels of employment among the perpetrators of neonaticide, in particular among those who killed female neonates. The lower level of employment among these perpetrators is not surprising as they were younger as well. We need to understand the role of poverty better, and qualitative data will provide more nuanced data on the context of such homicides.

We can safely assume that our estimate of the child homicide rate is an underestimate as child homicides have been described as a form of homicide that can easily be hidden. We consider this a limitation of the study. Among all categories of homicides, infanticide may be the most susceptible to misclassification, and it is difficult to differentiate infanticide from other causes of death during infancy, such as SIDS [17]. It is well known that a proportion of SIDS cases are concealed homicides [35]. Additionally, data from police were limited, and the motives and context for killing were not always identified, in particular for abandoned newborns. We therefore do not know whether children were abandoned with the intention that they would die or with the hope that someone would find them. We present only cases recorded in the mortuary system in 2009, and it is possible that there were some covert homicides that never entered the system; it is unlikely that we included non-homicide cases. We had a two-pronged approach for confirming homicides: through pathology records and through interviews with police detectives. We also very clearly excluded all stillbirths and deaths identified by the pathologist as SIDS cases. In addition, we included abandoned newborns/fetuses only if there was evidence of viability based on the baby’s mass (the average mass of included newborns was 2.5 kg) and estimated gestational age. Information on perpetrators, such as age and employment, was also not always known or recorded by police.

The killing of children is the extreme part of a continuum of violence against children in South Africa. A prevention focus should be the priority, but the current evidence base on prevention is limited. Interventions to prevent killings immediately after birth would be very different from interventions for older children, who are killed in different circumstances. One of the ways to identify risks and patterns and develop early intervention systems is to employ a child death review system similar to those used for prevention of maternal mortality and perinatal deaths (confidential enquiries) [36]. Such reviews could assist in identifying failures in the child protection system and assist in identifying cause of death in suspected abuse cases. A pilot child death review project has been initiated in South Africa in response to the first report from this study [5]. The process of developing review teams of all those involved in the investigation of child homicides has seen services come together for the first time [37]. Child death reviews over a number of years from the UK have shown the consistent identification of modifiable factors in almost two-thirds of the deaths reviewed [38], indicating a need to develop prevention and management strategies.

### Conclusion

This study shows that homicide of young children is a serious social and public health problem and suggests that abandonment of neonates is an indication of the failure of reproductive and other health services. Intervening is critical at all levels, including reproductive services as well as child protection services.

## Supporting Information

S1 STROBE checklist (DOC)

S1 Text (DOCX)

## Abbreviations

MLL: medico-legal laboratory
NIMSS: National Injury Mortality Surveillance System
SIDS: sudden infant death syndrome
