# Supplementary material for: Gender Differences in Homicide of Neonates, Infants, and Children under 5 y in South Africa: Results from the Cross-Sectional 2009 National Child Homicide Study
Source: PLoS Med. 2016 Apr 26;13(4):e1002003. doi: 10.1371/journal.pmed.1002003 (PMC4846035; doi:10.1371/journal.pmed.1002003)
Supplement: S1 STROBE checklist — (DOC) [file pmed.1002003.s001.doc]

STROBE Statement—checklist of items that should be included in reports of observational studies

|  | | Item No | Recommendation |
| --- | --- | --- | --- |
| **Title and abstract** | | 1 | Gender differences in homicide of neonates, infants and under-five year olds in South Africa: Results from the **cross-sectional** 2009 national child homicide study |
|  |  |  | (*b*) Provide in the abstract an informative and balanced summary of what was done and what was found  **Done- see title above** |
| Introduction | | | |
| Background/rationale | | 2 | Explain the scientific background and rationale for the investigation being reported  **Done - Introduction, paragraph 2-5: We explained conceptual hypotheses for describing the epidemiology (population rates/scope/size) of under 5 homicide with special attention to gender and age patterns.** |
| Objectives | | 3 | State specific objectives, including any prespecified hypotheses  **Done - Introduction, last paragraph** |
| Methods | | | |
| Study design | | 4 | Present key elements of study design early in the paper  **Done** |
| Setting | | 5 | Describe the setting, locations, and relevant dates, including periods of recruitment, exposure, follow-up, and data collection  **Done: Methods, paragraph 1** |
| Participants | | 6 | (*a*) *Cohort study*—Give the eligibility criteria, and the sources and methods of selection of participants. Describe methods of follow-up  **Done- Eligibility criteria explained in Methods paragraph 2-3.** |
|  |  |  | (*b*) *Cohort study*—For matched studies, give matching criteria and number of exposed and unexposed  **Not applicable** |
| Variables | | 7 | Clearly define all outcomes, exposures, predictors, potential confounders, and effect modifiers. Give diagnostic criteria, if applicable  **Done- Methods, paragraph 2-3 : variables extracted described** |
| Data sources/ measurement | | 8* | For each variable of interest, give sources of data and details of methods of assessment (measurement). Describe comparability of assessment methods if there is more than one group  **Done - Methods: data sources explained in same paragraphs as in 7 above** |
| Bias | | 9 | Describe any efforts to address potential sources of bias  **Not applicable – descriptive analysis** |
| Study size | | 10 | Explain how the study size was arrived at  **Done- Methods: paragraph 1 - sample size explained** |
| Quantitative variables | | 11 | Explain how quantitative variables were handled in the analyses. If applicable, describe which groupings were chosen and why  **Done- Methods: last paragraph- analysis took into account the cluster sample survey design** |
| Statistical methods | | 12 | (*a*) Describe all statistical methods, including those used to control for confounding  **Done - Methods: last paragraph** |
|  |  |  | (*b*) Describe any methods used to examine subgroups and interactions  **Done - Methods: last paragraph - domain analysis used described to analysis sub groups and testing of interactions between urban, rural , age and sex was done.** |
|  |  |  | (*c*) Explain how missing data were addressed  **Results, Table 2 missing data for characteristics were coded as unknown**  **Results Table 3: variables with missing data was identified with *** |
|  |  |  | (*d*) *Cohort study*—If applicable, explain how loss to follow-up was addressed  **Not applicable** |
|  |  |  | (*e*) Describe any sensitivity analyses  **Not applicable** |
| Results | | | |
| Participants | 13* | (a) Report numbers of individuals at each stage of study—eg numbers potentially eligible, examined for eligibility, confirmed eligible, included in the study, completing follow-up, and analysed  **Not Applicable**: | |
|  |  | (b) Give reasons for non-participation at each stage  **Not applicable** | |
|  |  | (c) Consider use of a flow diagram  **Not applicable** | |
| Descriptive data | 14* | (a) Give characteristics of study participants (eg demographic, clinical, social) and information on exposures and potential confounders  **Results, Table 2 &3** | |
|  |  | (b) Indicate number of participants with missing data for each variable of interest  **Results: Table 2 & 3** | |
|  |  | (c) *Cohort study*—Summarise follow-up time (eg, average and total amount)  **Not applicable** | |
| Outcome data | 15* | *Cohort study*—Report numbers of outcome events or summary measures over time  **Not applicable** | |
|  |  | *Case-control study—*Report numbers in each exposure category, or summary measures of exposure  **Not applicable** | |
|  |  | *Cross-sectional study—*Report numbers of outcome events or summary measures  **Results: Tables 1,2,3** | |
| Main results | 16 | (*a*) Give unadjusted estimates and, if applicable, confounder-adjusted estimates and their precision (eg, 95% confidence interval). Make clear which confounders were adjusted for and why they were included  **Results: Prevalence estimates with 95% CI presented in Table 2&3** | |
|  |  | (*b*) Report category boundaries when continuous variables were categorized  **Results: Age divided into 3 categories. See Table1-3** | |
|  |  | (*c*) If relevant, consider translating estimates of relative risk into absolute risk for a meaningful time period  **Not applicable** | |
| Other analyses | 17 | Report other analyses done—eg analyses of subgroups and interactions, and sensitivity analyses  **Methods – last paragraph: Subgroup analysis used as study part of a larger study of children- 1-18 years- see 12 above**  **Regression analysis used to test differences between means and logistic regression for differences between categorical data.** | |
| Discussion | | | |
| Key results | 18 | Summarise key results with reference to study objectives  **Discussion: paragraph 1** | |
| Limitations | 19 | Discuss limitations of the study, taking into account sources of potential bias or imprecision. Discuss both direction and magnitude of any potential bias  **Done - Discussion: paragraph 4 & 5** | |
| Interpretation | 20 | Give a cautious overall interpretation of results considering objectives, limitations, multiplicity of analyses, results from similar studies, and other relevant evidence  **Done- Discussion paragraph 5** | |
| Generalisability | 21 | Discuss the generalisability (external validity) of the study results  **Done- this is a national sample and one of the key outcomes is the national estimates of under year homicides** | |
| Other information | | | |
| Funding | 22 | Give the source of funding and the role of the funders for the present study and, if applicable, for the original study on which the present article is based  **Done** | |

*Give information separately for cases and controls in case-control studies and, if applicable, for exposed and unexposed groups in cohort and cross-sectional studies.

**Note:** An Explanation and Elaboration article discusses each checklist item and gives methodological background and published examples of transparent reporting. The STROBE checklist is best used in conjunction with this article (freely available on the Web sites of PLoS Medicine at http://www.plosmedicine.org/, Annals of Internal Medicine at http://www.annals.org/, and Epidemiology at http://www.epidem.com/). Information on the STROBE Initiative is available at www.strobe-statement.org.
