# Supplementary material for: Gender Differences in Homicide of Neonates, Infants, and Children under 5 y in South Africa: Results from the Cross-Sectional 2009 National Child Homicide Study
Source: PLoS Med. 2016 Apr 26;13(4):e1002003. doi: 10.1371/journal.pmed.1002003 (PMC4846035; doi:10.1371/journal.pmed.1002003)
Supplement: S1 Text — (DOCX) [file pmed.1002003.s002.docx]

**Title**: Gender differences in homicide of neonates, infants and under-five year olds in South Africa: Results from the cross-sectional 2009 national child homicide study

**Analysis plan**

The analysis of the homicides of a children under 5 years was motivated by the need to develop a hypothesis on the gender and age patterns of this group and the absence of such analyses in the literature globally [1]. This analysis and description of the epidemiology of under 5 year old homicide was based on our previous work of all child homicides[2] and the two female homicide studies[3]. This current analysis is therefore a sub analysis of a repeat study and the data analysis was therefore well known to the authors.

However children was not included in the 1^st^ study and the plan of the present study was designed in November 2014 when Naeemah Abrahams presented her plan to the co-investigators to do specific analysis of gender and age patterns among the under 5 year age group. The 1^st^ paper on all child homicides from the database revealed the under 5 year age group as the second most at risk group. Initial analysis also revealed that the homicide pattern and scope of the neonates have not been described at national level.

Nadine Nannan a demographer on child death in South Africa was approached to assist with the calculation of population rates and these are described in Methods last paragraph.

The full analysis process is explained in the manuscript ‘Data were analysed using Stata version 13. The data analysis took into account the cluster sample survey design and weights were applied to account for the selection probabilities of Medical Legal Laboratories (MLL). The survey included homicides of all children up to 18 years and we used domain analysis which allowed us to analyse the subpopulations of interest i.e. subgroup children under 5 years and the subgroup of abandoned neonates. Descriptive statistics (survey means and proportions) were calculated, as well as standard errors and 95% confidence intervals. Categorical variables were compared using Pearsons *χ*2 test and standard errors and 95% confidence intervals (CIs) were calculated using methods for complex sample surveys (Taylor linearization). We used a regression analysis to test differences between means for continuous variables and logistic regression for differences in proportions for categorical data. We were particularly interested to test if age and sex of the child were associated with a number of outcomes (urban rural status, child abuse and perpetrator age) and the models were tested with interactions terms. We stratified the ages of the children into early neonates (0-6 days), late neonates (7-28 days), 1-12 months and 1-4 years to do age specific analysis (Table 2). Live births derived from the 2011 Census were used to calculate the age-specific mortality occurring in the first year of life. The mortality rates of children aged 1 to 4 years old were calculated using an alternative to the official set of mid-year population estimates produced by the Centre for Actuarial Research (Table 1) . We also performed a subpopulation analysis for the abandoned neonates and looked at differences between boys and girls.’

1. Fund, U.N.C., *Hidden in plain sight: A statistical analysis of violence against children*. 2014, United Nations Children's Fund New York.

2. Mathews S, Abrahams N, Jewkes R, Martin LJ, Lombard C. The Epidemiology of Child Homicides in South Africa. WHO Bulletin. 2013;91:562-8. Epub 31 May 2013. doi: doi: <http://dx.doi.org/10.2471/BLT.12.117036>.

3. Abrahams N, Mathews S, Martin LJ, Lombard C, Jewkes R. Intimate Partner Femicide in South Africa in 1999 and 2009. PLoS Medicine. 2013;10(4):e1001412.
